# Synthetic 1,4-Pyran Naphthoquinones Are Potent Inhibitors of Dengue Virus Replication

**DOI:** 10.1371/journal.pone.0082504

**Published:** 2013-12-20

**Authors:** Emmerson C. B. da Costa, Raquel Amorim, Fernando C. da Silva, David R. Rocha, Michelle P. Papa, Luciana B. de Arruda, Ronaldo Mohana-Borges, Vitor F. Ferreira, Amilcar Tanuri, Luciana J. da Costa, Sabrina B. Ferreira

**Affiliations:** 1 Laboratório de Genômica Estrutural, Instituto de Biofísica Carlos Chagas Filho, Universidade Federal do Rio de Janeiro, Rio de Janeiro, Rio de Janeiro, Brazil; 2 Universidade Federal do Rio de Janeiro, Departamento de Virologia, Instituto de Microbiologia Paulo de Góes, Rio de Janeiro, Rio de Janeiro, Brazil; 3 Universidade Federal Fluminense, Departamento de Química Orgânica, Instituto de Química, Niterói, Rio de Janeiro, Brazil; 4 Universidade Federal do Rio de Janeiro, Departamento de Genética, Instituto de Biologia, Rio de Janeiro, Rio de Janeiro, Brazil; 5 Universidade Federal do Rio de Janeiro, Departamento de Química Orgânica, Instituto de Química, Macaé, Rio de Janeiro, Brazil; University of Minnesota, United States of America

## Abstract

Dengue virus infection is a serious public health problem in endemic areas of the world where 2.5 billion people live. Clinical manifestations of the Dengue infection range from a mild fever to fatal cases of hemorrhagic fever. Although being the most rapidly spreading mosquito-borne viral infection in the world, until now no strategies are available for effective prevention or control of Dengue infection. In this scenario, the development of compounds that specifically inhibit viral replication with minimal effects to the human hosts will have a substantial effect in minimizing the symptoms of the disease and help to prevent viral transmission in the affected population. The aim of this study was to screen compounds with potential activity against dengue virus from a library of synthetic naphthoquinones. Several 1,2- and 1,4-pyran naphthoquinones were synthesized by a three-component reaction of lawsone, aldehyde (formaldehyde or arylaldehydes) and different dienophiles adequately substituted. These compounds were tested for the ability to inhibit the ATPase activity of the viral NS3 enzyme in *in vitro* assays and the replication of dengue virus in cultured cells. We have identified two 1,4-pyran naphthoquinones, which inhibited dengue virus replication in mammal cells by 99.0% and three others that reduced the dengue virus ATPase activity of NS3 by two-fold in *in vitro* assays.

## Introduction

Dengue viruses belong to the *Flaviviridae* family and include four antigenic serotypes (DENV-1, DENV-2, DENV-3 and DENV-4) [1–3]. Human infection by any of DENV serotypes may cause a spectrum of clinical manifestations ranging from mild dengue fever to the severe forms of dengue hemorrhagic fever (DHF) and dengue sock syndrome (DSS), which can be fatal [1–3]. DENV is transmitted by *Aedes* mosquitoes present in tropical and subtropical areas in the world, where at least 2.5 billion people live [1,2]. According to the World Health Organization, the infection affects over a 100 million people annually and dengue is considered one of the most severe arthropod-borne disease and a substantial public health problem [1,2].

Infection by one DENV serotype elicits long-term protection against that particular serotype but not against the others [4]. In addition, sequential exposure to more than one serotype increases the risk for the development of severe dengue [4]. Current preventative measures are almost exclusively based on mosquito control programs, which alone have not been successful in controlling the spreading of the infection [5]. The development of an effective vaccine is under investigation; however, it’s been hampered by viral antigenic variation and insufficient knowledge of the mechanisms by which human beings are protected against infections with the different DENV serotypes [4]. In this regard, a tetravalent chimerical anti-DENV vaccine was recently enrolled in a phase 2b clinical trial and reached only 30.2% overall effectiveness, with no significant protection against DENV-2 [6].

Therefore, the search for natural or synthetic substances with specific antiviral activity without toxicity to normal cells in humans is a desired strategy to avoid severe dengue and help controlling dengue dissemination [7]. The various stages of the viral life cycle represent individual therapeutic targets that can be exploited; however, few antiviral drugs have been tested until now and little is known about their biological effects [7]. 

Non-structural DENV proteins, which have well defined enzymatic activities, are the most promising targets to the development of anti-DENV compounds. The non-structural protein 3 (NS3) is a multifunctional enzyme that has serine protease activity in the protease domain (located at the N-terminus of NS3), and NTPase, Helicase and RTPase activities in the helicase domain (located at the C-terminus of NS3). These activities are essential in the process of replication and capping of RNA viruses [8,9]. The helicase domain promotes the hydrolysis of ATP as a source of energy for the dissociation of double stranded RNA replication intermediates [9]. The cleavage of the full-length viral polyprotein between NS2A-NS2B, NS2B-NS3, NS3-NS4A, NS4A-NS4B and NS4B-NS5 boundaries is mediated by the serine protease domain of NS3, which uses a hydrophobic segment of 40 residues of NS2B (NS2BCF40) that is an essential cofactor for the NS3 proteolytic activity [10,11]. These activities are considered essential for the viral replication process. 

Based on studies of the NTPase/helicase domains of the NS3 of HCV, the major obstacle in the development of inhibitors for these domains are associated with conformational changes of sub domains 1 and 2 [12] that lead to low specificity of the inhibitors that bind in the NTPase site and in the cleft of dissociation of RNA [13]. However, unlike the ATPase site of NS3 of HCV, which presents the problem above, the ATP cleft of the interaction of DENV NS3 may be a promising target since the NS3 protease domain resides next to the entrance of the ATPase active site between the helicase sub domains 1 and 2 [14,15]. 

**Figure 1:**
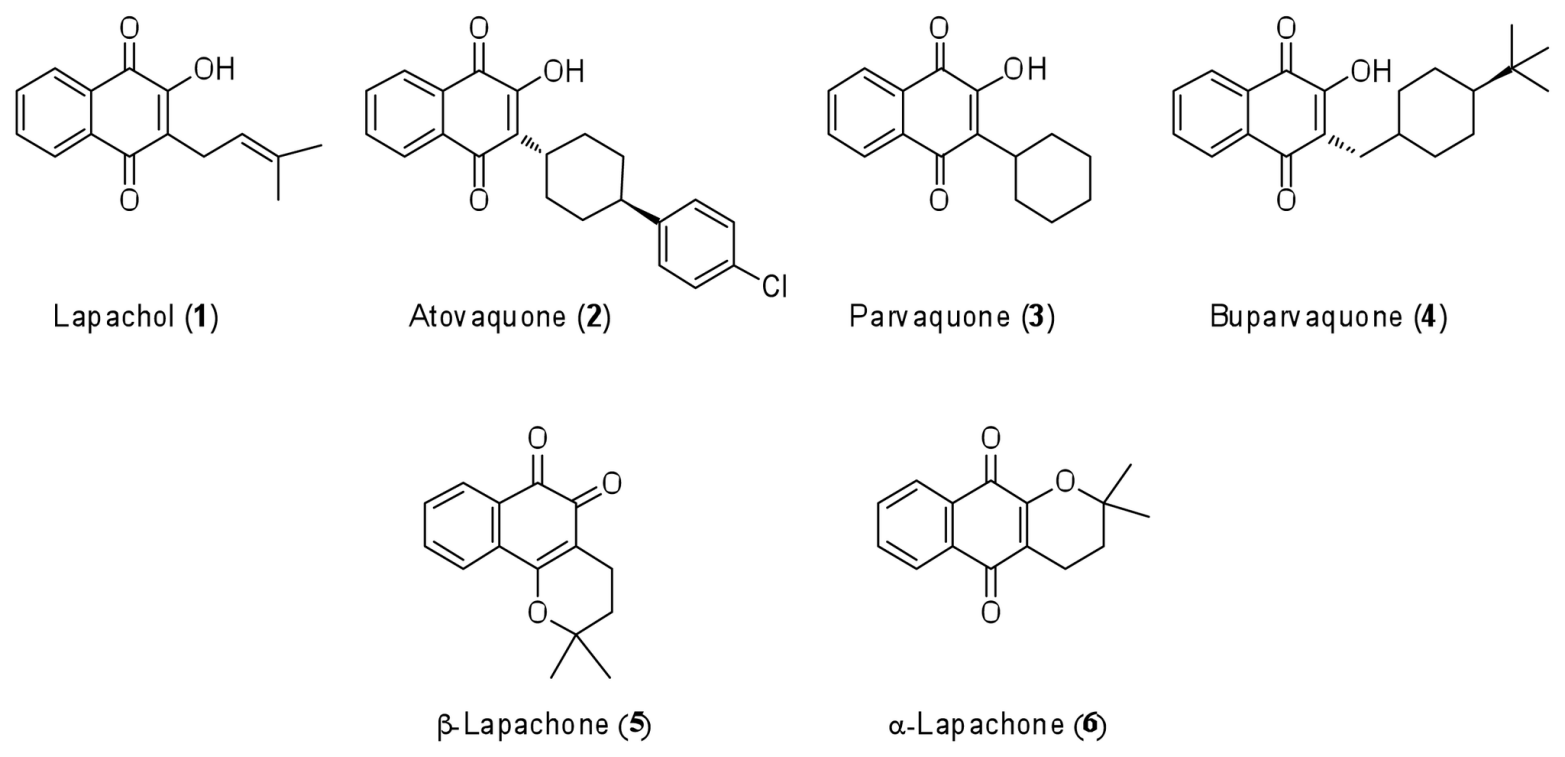
Natural and synthetic bioactive naphthoquinones. Shown are Lapachol (1), Atovaquone (2), Parvaquone (3), Buparvaquone (4), β-Lapachone (5) and α-Lapachone (6).

The natural naphthoquinones have different biological activities and some compounds of this class such as vitamins K-type, mitomycin, and anthracyclines came to the industrial production and clinical use as drugs for a number of diseases. Amongst the group of natural naphthoquinones, lapachol (1) is the best-known member (Figure 1). It occurs as a component of the various plant families, including the *Bignoniaceae*, *Leguminosae*, *Sapotaceae*, *Scrophulariaceae*, *Verbenaceae*, *Malvaceae*, and *Proteaceae* and exhibits an impressive list of noteworthy biological activities such as: trypanocidal [16–18]; antitubercular [19]; antibacterial [20]; antimalarial [21]; pesticidal; antitumoral; [22–24]; anti-leishmanial [25–27]; activity against snail *Biomphalaria glabrata* that is involved in the transmission of schistosomiasis [28,29], among others [30]. Its structure has been used as a base for other similar pivotal 3-substituted-2-hydroxy-1, 4-naphthoquinone as atovaquone (2), parvaquone (3) and buparvaquone (4) that are key drugs used for the treatment of *Pneumocystis* pneumonia, toxoplasmosis and malaria. The examples above highlight the importance of this class of compounds.

Two natural pyran naphthoquinones isomers of lapachol (1) - β-lapachone (5) and α-lapachone (6) have significant biological activity that has been widely explored and their structures used as template for development of new synthetic naphthoquinones [19]. These two substances can also be easily obtained from lapachol. The pyran naphthoquinone (5) had also showed beneficial biological activity as antibacterial and antifungal [31], trypanocidal [32], anticancer [33,34] and antiviral [35]. The mechanism of action of pyran naphthoquinones is not entirely elucidated despite the broad range of biological activities of these molecules. Some studies suggest that they are active at the level of the nuclear enzymes topoisomerases I and II, which are essential for chromosome structure, DNA transcription, and replication [36,37]. Other authors point out that the biological profiles of these substances are due to their ortho or para-quinonoid moiety that can accept one and/or two electrons creating a redox cycling, which generates radical anion, superoxide anion radical, or dianion species, leading to an intracellular deleterious hypoxic condition [38].

In this study, we demonstrated that 1,4- pyran naphthoquinones are potent inhibitors of DENV-2 replication in cells and impact on the *in vitro* ATPase activity of NS3.

## Materials and Methods

### Synthesis of the pyran naphthoquinones

**Figure 2:**
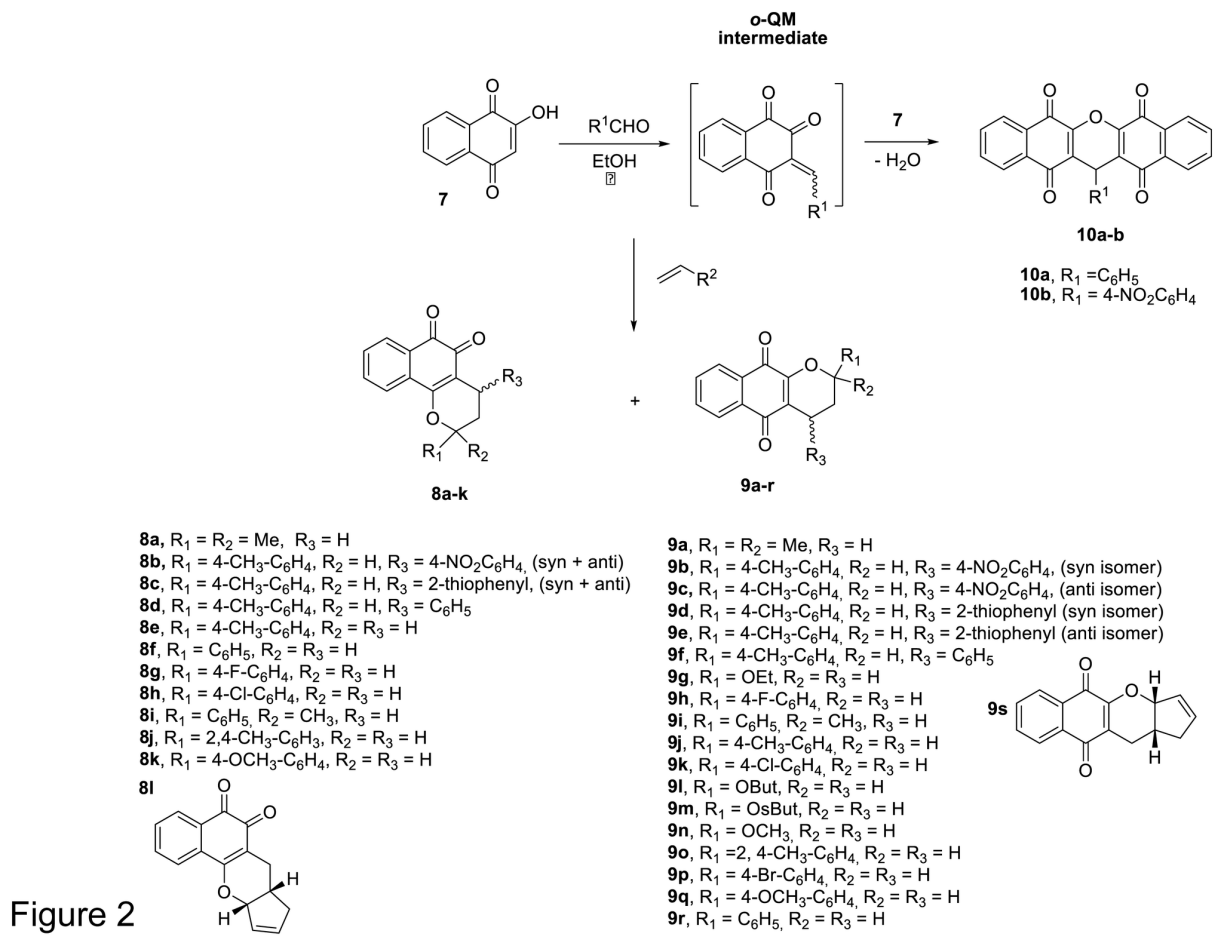
General scheme used for preparation of pyran naphthoquinones. The o-QM intermediate is generated in situ by Knoevenagel condensation of 2-hydroxy-1,4-naphthoquinone (7) with formaldehyde and arylaldehydes. The subsequent hetero Diels-Alder reaction with substituted dienophiles in aqueous ethanol media yields high amounts of α- and β-lapachone derivatives.

The methodology for synthesis of the pyran naphthquinones used in this study has been reported elsewhere [39,40]. Briefly, the compounds were obtained by reacting of lawsone (7, 2-hydroxy-1,4-naphthoquinone) with an appropriate aldehyde (formaldehyde or arylaldehydes) that generate in situ an o-quinone methide intermediate (o-QM, via the Knoevenagel condensation, which then undergoes a hetero Diels-Alder reaction with an appropriate dienophiles. The xanthenes 1a and b were produced by nucleophilic addition of lawsone (7) on o-quinone methide intermediate followed by dehydration (Figure 2). The scope of this three-components reaction is not limited of any aldehyde. At the end of the reaction the products were purified by column chromatography using silica gel and its characterization by spectroscopic techniques was previously published [39,40].

### Construction of full-length NS3 expression plasmid

The plasmid pRS424-FLDEN2-NG-CDNA, containing the full-length DENV-2 genome from the New Guinea strain, was used as template for the generation of the full-length NS3 (NS3FL) construct. The fragment corresponding to amino acids 1 to 619 of NS3FL was amplified using the primers NS3Pro_BamHI-F (5’-AATAggATCCgCTggAgTATTgTgggATgT-3’) that incorporates a *Bam* H I restriction site at the 5´ terminus) and NS3hel_KpnI-R (5’-AATAggTACCCTACTTTCTT CCAgCTgCAAACTC -3´) that incorporates a *Kpn* I restriction site and stop codon (in bold) at the 3’ terminus. PCR reaction were performed in 1X PCR buffer (Invitrogen – Carlsbad, USA), 1.5 mM of MgSO4 (Invitrogen – Carlsbad, USA), 125 µM of each dNTP, 50 pmol of each primer and 1.25 U of Pfx DNA polymerase (Invitrogen – Carlsbad, USA) with 100-200 ng of genomic DNA in a final volume of 50 µL; amplification conditions being 94 °C (4 min), followed by 35 cycles of 94 °C (1 min), 60 °C (1 min), and 68 °C (2 min). The PCR products were purified with a GFX PCR DNA and gel band purification kit (GE-Healthcare, USA). The fragments were digested with BamH I (NEB, USA) and Kpn I (NEB, USA) and cloned into the pET21d vector modified for expression of 10xHis tag plus ubiquitin-site at the N-terminus. The authenticity of the construct was confirmed by sequencing.

### Expression and purification of full-length NS3 protein

*E. coli* cells (Rosetta [λDE3] strain) were transformed with the vectors containing the full-length NS3 (NS3FL) fusion with 10X histidine-ubiquitin at the N-terminus. Overnight cultures of the transformed cells were diluted 1/100 in 2 liters of LB medium containing 100 μg/mL ampicilin (USB, USA) and 34 μg/mL chloramphenicol (USB, USA) at 37 °C and grown until O.D._600nm_ reached 0.8. Next, the culture was cooled down to 25 °C and inducted for 4 hours after the addition of 0.5 mM isopropyl-1-thio-β-D-galactopyranoside (IPTG), under shaking (200 rpm). The cells were then harvested by centrifugation at 4,200 g for 20min and the pellet was stored at -20°C until used. 

For protein purification, precipitated cells were thawed and re-suspended in 25 mL buffer/L of original culture of buffer A (50 mM Tris-HCl pH 8.0, 500 mM NaCl, 5 mM β-mercaptoethanol), and lysed by adding 5 mg/mL lysozyme (Sigma) and 20 µg/mL DNase. The cell lysates were centrifuged at 27,000 g for 40 min at 4 °C and the pellet was re-suspended in 50 mL of buffer B (50 mM Tris-HCl, pH 8.0, 500 mM NaCl, 5 mM β-mercaptoethanol, 8 M urea, and 10% glycerol), centrifuged as before and the supernatant solution was purified chromatographically using 40 mL Ni^2+^-NTA affinity resin (Qiagen) that was pre-equilibrated with 180 mL buffer B. The column was extensively washed with buffer B, and protein was then eluted from the column with a crescent imidazole gradient (0 to 250 mM). The urea-solubilized NS3FL was removed by stepwise dialysis with 30 volumes of buffer C (50 mM Tris-HCl, pH 8.0, 500 mM NaCl, 5 mM β-mercaptoethanol, 0.05% CHAPS, and 10% glycerol) for 16 h at 4 °C. After protein refolding by dialysis, the 10His+ubiquitin site was excised with yeast ubiquitin hydrolase (YUH), producing only the NS3FL. The sample was again applied onto a Ni^2+^-NTA affinity column to remove the 10xHis+ubiquitin and YUH, centrifuged at 27,000 g for 40 min at 4 °C and the NS3FL protein (without 10XHis-ubiq tag) was dialyzed against buffer C. The concentration of the NS3FL was determined spectrophotometrically using molar extinction coefficients of 69339.3 M^-1^cm^-1^. The protein was stored at – 80 °C. 

### In vitro Colorimetric NTPase assay and inhibition measurement

The ATPase activity of NS3 was determined by measuring the extension of hydrolysis of NTP to NDP and Pi. The amount of free inorganic phosphate released was calculated by the hydrolysis of ATP using a standard curve with known Pi concentrations, and the reaction was measured at 660 nm using SpectraMax M5 spectrophotometer (Molecular Devices). Compounds were diluted from 20 mM or 40 mM stock solution prepared in 100% DMSO. These compounds were diluted to a final concentration of 10% DMSO in the assay buffer. The compound screening assay was performed in 96-well plate, and in each well reaction containing 600 nM NS3FL, 40 mM Tris-HCl (pH 7.5), 5 mM MgCl_2_, 5 mM DTT, 0.1M KCl were pre-incubated with 50µM of compounds (or varying concentrations of the compound) for 10 min at 30 °C. The kinetic activity was initiated by the addition of 1 mM of ATP substrate and incubation for 10 min at 30°C. Reactions were stopped with addition of 20 μL 50% TCA, followed by 80 μL of ammonium molibidate solution and 40 μL of reducing agent [41]. Absorbance was read and data were processed on Sigma Plot Version 10.0. The initial reaction velocities (V_i_) of product formation were determined from progress curves using the linear regression method and data converted to residual activity and/or calculate IC_50%_ assuming Michelis-Mentem kinetics. The experiments were carried out in duplicate and the statistical difference between the inhibition reaction in the presence of each compound and the control were evaluated by unpaired t-test analysis.

### Cells and virus stocks

Vero (kidney epithelia from African Green Monkey) and HepG2 (human hepatocellular carcinoma) cells were obtained from the ATCC (Manassas – VA, USA), and maintained in Dulbecco's Modified Eagle Media supplemented with 5% Fetal Calf Serum and antibiotics. Cells were passage each 3-4 days in a concentration of 4x10^4^ cells/cm^2^ and assayed in 96 well plates at a concentration of 1x10^4^ plated the day before.

Viruses used in this work include a DENV-2 prototype 16681 and a Brazilian isolate (44/02 - from Fundação Oswaldo Cruz – RJ - Brazil). Virus stocks were generated by infecting Vero cells at an 80% confluence with a multiplicity of infection (m.o.i) of 0.1. Infected cells were incubated at 37 °C for 5-7 days and inspected every day until the cytopathic effect was clearly observed. Supernatants were then collected, centrifuged at 1,200 x g for 10 min. to clear cellular debris and stored at -70°C until use. The titter of viral stocks was measured by TCID(50)% using serial dilutions of 10^1^ to 10^7^ of the viral stocks inoculated into Vero cells. The viral titter was calculated according with Reed and Munch [42]. 

### Cytotoxicity Assay

Cell toxicity of all compounds used in this work was tested in Vero and HepG2 cells by Neutral Red dye exclusion assay. Briefly, cells were cultured with varying concentrations of each compound and incubated for 72 hours at 37°C. After this period, the cells were incubated with 0.05% neutral red solution for 3 hours, washed with 1X PBS and fixed with 20% paraformaldehyde for 5 min. Cells were washed again and the incorporated dye was extracted with a 50% methanol and 1% acetic acid solution for 20 min. Plates were read at 490nm in a ELISA reader. Results obtained with the neutral red dye exclusion assay were confirmed using the CellTiter Blue Cell Viability Kit (Promega, Madison - USA) according to manufacturer’s instructions. 

### Virus Replication Inhibition Assay

In order to test the anti-viral effect of the compounds used in this work, monolayers of Vero cells were inoculated with DENV-2 at an m.o.i of 0.1 for 2 hours. After this period, cells were washed with 1X PBS to remove unbound viruses and fresh media was added with the highest non-cytotoxic concentration of the compounds. Infected cells were also cultured with culture medium or 1% DMSO, as negative controls, or with 200μM of Ribavirin® as reference drug for DENV replication inhibition. After 72 h, culture supernatants were collected and viral RNA was extracted using the QIAamp Viral RNA Mini Kit (Qiagen – Hamburg, Germany), according to manufacturer’s instructions. The synthesis of cDNA was performed with random hexamers as primers and the High Capacity cDNA kit (LifeTechnologies – Carlsbad, USA) according to manufacturer’s instructions. The detection and quantification of the cell free viral genomic RNA was performed by qPCR using specific primers and probe hybridizing in the protein E gene region as already described [43]. qPCR conditions were as follow: a reaction mixture consisting of 12.5μL of TaqMan (Invitrogen – Carlsbad, USA), 50 pmol of each primer, 9 pmol of the probe and 7μL of the cDNA reaction to a final volume of 25μL was submitted to 40 cycles of amplification in a ABI PRISM 7000 Detection System (LifeTechnologies – Carlsbad, USA) using 60°C annealing temperature. Amplification results were analyzed using the 7000 SDS software (LifeTechnologies – Carlsbad, USA) and the threshold parameter was manually adjusted. 

The efficiency of the qPCR was calculated based on the slope of a Ct versus Template Concentration curve. To generate the calibration curve, a purified stock of DENV-2 containing 10^5^ Plaque forming unities/ml was serially diluted and used to RNA extraction followed by cDNA synthesis, which was in turn used as template for the qPCR. A value of -3.30 was achieved, which represents an efficiency close to 100%. Based on this efficiency for the qPCR, the percentage of inhibition of DENV replication was calculated by the ΔΔCT method. 

A dose response curve was also performed with the compounds that inhibited DENV replication by at least 30%. For this purpose, DENV-2-infected Vero cells were cultured with the selected compounds at concentrations varying from 100 to 0.4 μM and virus replication was measured by qPCR as above. IC_50_ values were obtained by the Hill’s regression curve using the software Prism 5. Experiments were always performed in triplicates. All results were confirmed in HepG2 cells by qPCR and by TCID_50_ in Vero cells.

## Results and Discussion

### Drug screening

**Figure 3:**
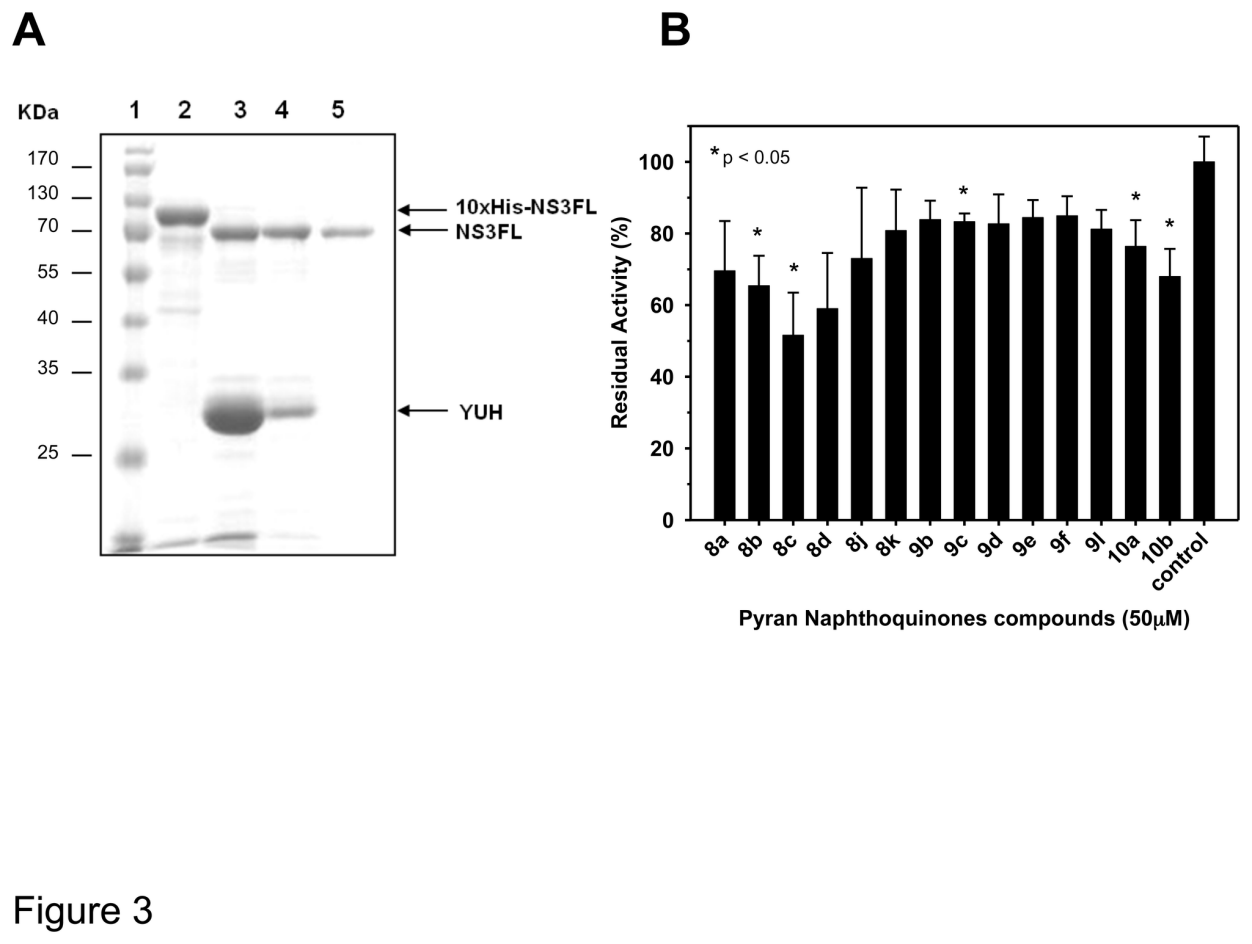
Inhibition of the ATPase activity of purified NS3 by naphthoquinones. (A) NaphSDS-Page analysis of the purification full-length NS3 (NS3FL) from *E coli*. Lane 1, molecular weight marker; lane 2, 10xHis-NS3FL (10xHis-Ubiquitin site) after refolding by dialysis; lane 3 and 4, NS3FL after 10xHis-Ubiquitin site tag excision by YUH in ratio 1:5 and 1:1, respectively; and lane 5, purified NS3FL protein following His-TRAP column chromatography. The gel was stained with Comassie brilhant blue G. (B) A range of pyran naphthoquinones was tested for their activity against ATPase site. The screening was performed in buffer containing 40 mM Tris-HCl (pH 7.5), 5 mM DTT, 100 mM KCl, and 5 mM MgCl_2_. The compounds were pre-incubated with 600nM NS3FL for 10 min at 30 °C followed by the addition of 1 mM ATP. The screening was carried out in duplicate and the unpaired t-test was used for the statistical analysis. Those compounds showing statistical difference from the control (p < 0.05) were labeled with an asterisk.

Our search for chemical compounds capable of inhibiting the ATPase activity of DENV-2 NS3 was based on the cloning, expression and purification by chromatographic methods of the full-length NS3 protein (NS3FL) for subsequent screening of pyran naphthoquinones. Figure 3A shows the amount of expression of purified NS3FL. The ATPase activity was determined at different concentrations of NS3FL in the presence of 2 mM ATP, whose conversion to ADP plus Pi was equivalent to the amount of protein in each reaction (data not shown). Moreover, the Michaelis constant of NS3FL was determined by measuring the ATPase activity of 200 nM of enzyme (in the absence of inhibitors and nucleic acid) at ATP concentrations from 0.25 to 3mM for 12min. The K_M_ and K_cat_ values of NS3FL were 0.524±0.058 mM and 0.157± 0.005474 min^-1^, respectively.

Once the ATPase activity was confirmed after purification process, we used the colorimetric assay developed by Fiske and Subbarow [41] for the screening of compounds, which was performed using 600 nM of NS3FL in reactions containing 40 mM Tris-HCl (pH 7.5), 5 mM DTT, 100 mM KCl, 5 mM MgCl_2_, and 1 mM ATP and 50 µM of the pyran naphthoquinones 8, 9 and 10. Fourteen pyran naphthoquinones were shown to be capable of inhibiting the ATPase activity of NS3. The results of the most effective pyran naphthoquinones are summarized in Figure 3B. The percentage of NS3FL ATPase activity inhibition varied from 15 to 50% of the untreated control. However, only the 1,2- pyran naphthoquinones 8b, 8c, and the 1,4-pyran naphthoquinones 9c, 10a, and 10b showed to be statistically different (p < 0.05). It is worthy note that compound 8c presented the highest inhibitory effect achieving two-fold reduction of the NS3 ATPase activity while 1,4-pyran naphthoquinones 9 were the least active (Figure 3B). 

### Inhibition of DENV-2 replication in Vero cells by Pyran Naphthoquinones

Given that all of the type 9 (b-f), 10 (a and b) and, mostly, type 8 pyran naphthoquinones showed inhibitory effect on the NS3 ATPase activity the effect of these substances on DENV-2 replication in cultured cells was evaluated. The compounds were initially screened regarding their toxicity level after culturing Vero and HepG2 cells with the effective concentration of each compound. After 72h, cellular viability was analyzed by neutral red staining and confirmed by the conversion of redox resazurin into fluorescent resorufin. All the compounds that showed less than 10% toxicity were selected for further analyzes (data not shown). 

**Figure 4:**
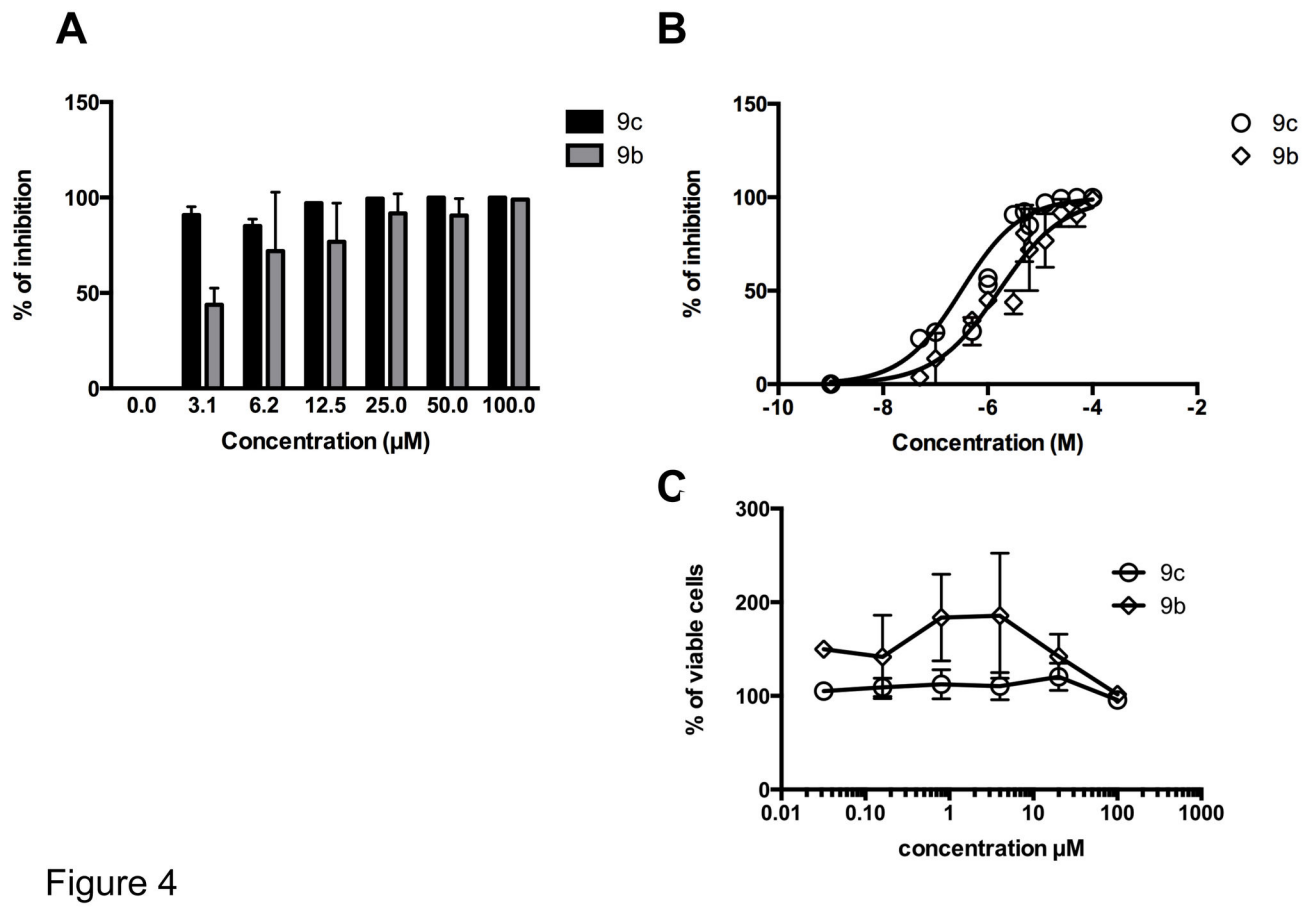
Dose-response curves and citotoxicity of 1,4-pyran naphthoquinones 9b and 9c in DENV-2-infected Vero cells. Vero cells were infected with DENV-2 at an m.o.i of 0.1 in the presence of increasing concentrations of the 1,4-pyran naphthoquinones 9b and 9c. 72 hours after infection the cell-free supernatants were harvested and processed for the quantification of produced viral progeny by qPCR. (A) The percentage of inhibition was calculated using the ΔΔCT values. (B) The concentration of each compound that inhibit 50% of the viral replication (IC_50_) was determined by a Hill’s regression curve after logarithmic interpolation of the data using the GraphPad 6 software. (C) The potential cytotoxic effect of each compound was evaluated in uninfected cells by the vital neutral red staying and represented as percentage of the untreated control. Data represent mean values ± standard deviations (SD) for three independent experiments.

To analyze the impact of the selected compounds on DENV replication, cells were infected and cultured with the highest non-toxic concentration of each compound and virus replication was analyzed by qRT-PCR. In our screening assay, we found that only compounds 9b and 9c were successful in blocking DENV-2 replication, achieving 99.0 and 99.6% of inhibition, respectively, with the 100 µM concentration (Figure 4A and Table 1). These compounds did not reduce the viability of uninfected host cell cultures, even at the 100 µM concentrations (Figure 4C). The inhibitory effect of these compounds on DENV-2 replication was confirmed by the Tissue Culture Infectious Dose assay (TCID_50_). In this assay DENV-2 replication was inhibited by 99.83% and 99.99% when compounds 9b and 9c, respectively, were used at a 100 µM concentration (Table 1). The differences in the percentage of inhibition when viral replication was measured by qRT-PCR and TCID_50_ were probably due to the differences in the sensibility of these assays. Ribavirin, used as a reference drug, inhibited DENV-2 replication by 78% at a concentration of 200μM when measured by the qRT-PCR assay (Table 1). These results demonstrate the remarkable efficacy of these 1,4- pyran naphthoquinones. 

**Table 1:** Inhibitory effect (%) of pyran naphthoquinones on DENV-2 replication.

Pyran naphthoquinone (μM)	% of Inhibition of DENV-2 Replication (qPCR)*	% of Inhibition of DENV-2 Replication (TCID_50_)*
8c (0.4)	NT	NT
8d (25)	0	NP
9b (100)	99.0	99.83
9c (100)	99.6	99.99
9d (0.4)	NT	NT
9e (0.4)	NT	NT
9f (0.4)	NT	NT
10a (12.5)	0	NP
10b (12.5)	0	NP
Ribavirin (200)	78.00	NT

^*^ Screening assays were performed with qPCR for quantification of the produced viral progeny in DENV-2 infected Vero cells at the highest non-toxic concentration of the pyran naphthoquinone compounds, followed by confirmation with Tissue Culture Infectious Dose of 50 (TCID_50_) assays. For those compounds that did not show inhibition in the qPCR assay the TCID_50_ assay was not performed (NP).

NT- not tested for viral replication due to its high cytotoxicity both in Vero and HepG2 cells.

The most active compound *in vitro* (8c), which is a 1,2- pyran naphthoquinone, was highly toxic to the cell culture even at a concentration as low as 0.4 μM, and it was no further tested against viral replication. In addition, the 1,2, and 1,4- pyran naphthoquinones, respectively 8d, and 10b, which were also active *in vitro*, did not inhibit DENV-2 replication (Table 1).

The replication of DENV-2 in Vero cells was inhibited in a dose-dependent way for compounds 9b and 9c (Figure 4A). We then determined the IC_50_ of these compounds based on the non-linear regression of the dose-response curve (Figure 4B). The IC_50_ values were 0.3114 μM and 1.644 μM for the 1,4-pyran naphthoquinone 9c and 9b, respectively (Figure 4B). The IC_50_ for the 1,4-pyran naphthoquinone 9c was 5.3-fold lower than that of the compound 9b, demonstrating that compound 9c is the most potent in inhibiting viral replication (Figure 4A and C, respectively). Importantly, both compounds showed much higher inhibition levels in cell culture than on the NS3 ATPase activity *in vitro*, suggesting that they may have other effects influencing on virus replication. 

Moreover, two new synthesis of each compound (9b and 9c) were made and showed similar inhibitory activity against DENV-2 replication, indicating that the antiviral activity of these compounds is reproducible.

### Dose response effect of pyran naphthoquinones in inhibition of NS3 ATPase

**Figure 5:**
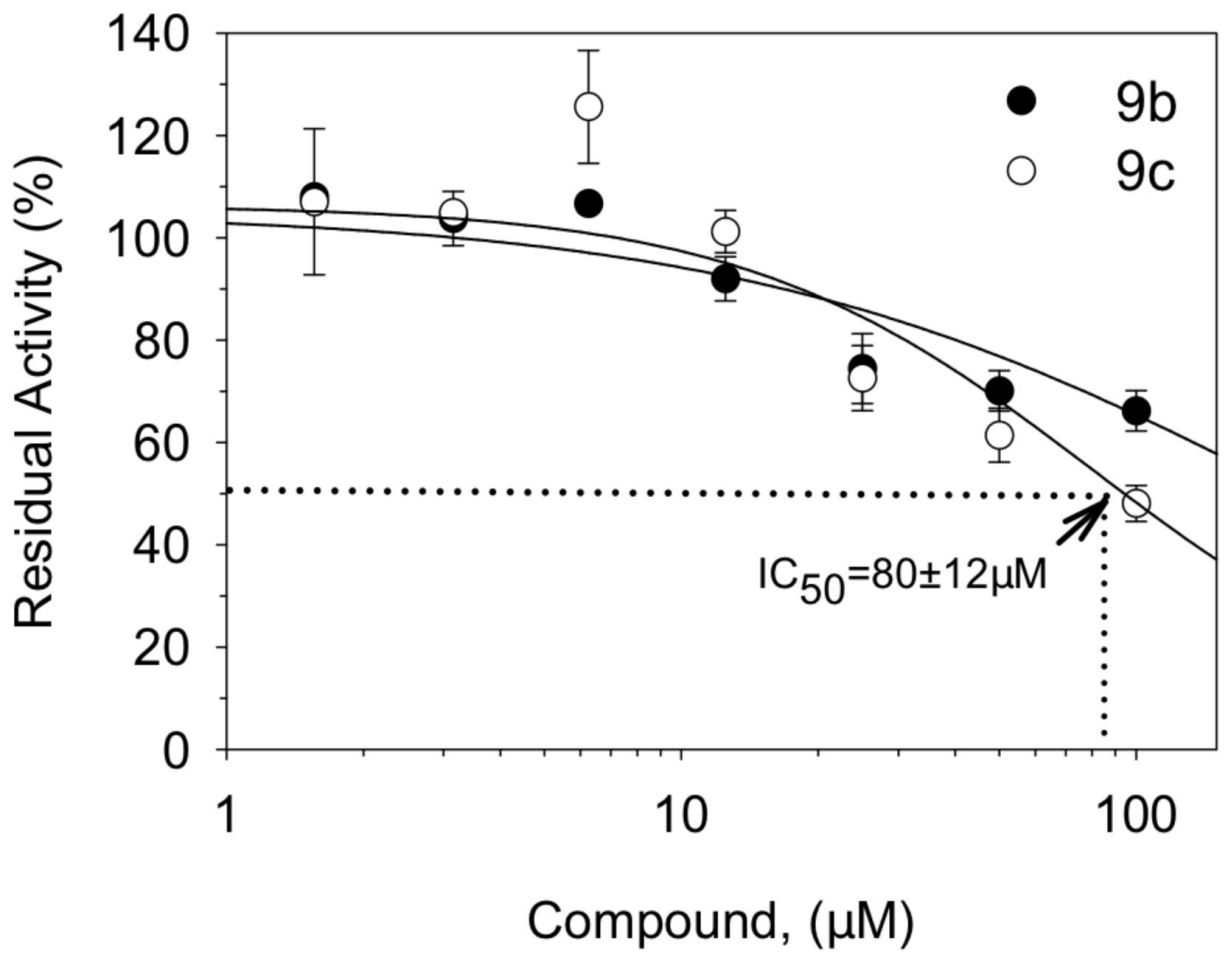
Inhibition of the ATPase activity of full-length NS3 by the compounds 9b and 9c. Increasing concentrations of naphthoquinones 9b and 9c were tested for their activity against ATPase site. The reaction was performed in buffer containing 40 mM Tris-HCl (pH 7.5), 5 mM DTT, 100 mM KCl, and 5 mM MgCl_2_. The compounds were pre-incubated with 600nM NS3FL for 10 min at 30 °C followed by the addition of 1 mM ATP. Each point represents the average of three independent replicates in different concentrations of the compounds.

Since the compounds 9b and 9c demonstrated specific inhibitory activity in DENV-2 replication in Vero cells, we further investigated their efficacy against NS3 ATPase and helicase activities by performing a dose response analysis. Due to the low solubility of the naphthoquinone compounds in buffer reaction, the ATPase assay was performed at concentrations ≤ 100 μM. In this condition, the compound 9c (IC_50_ = 80±12 μM) showed a higher inhibitory effect of the NS3 ATPase activity than the compound 9b (IC_50_ > 100μM) (Figure 5). The difference observed in the concentration required to inhibit 50% of the NS3 ATPase activity for the naphthoquinones 9c and 9b is in agreement with the difference in the IC_50_ values obtained for the DENV-2 replication in cells. Again, the naphthoquinone 9c was more effective in inhibiting both the NS3 ATPase activity and the replication of DENV-2 in Vero cells than the naphthoquinone 9b. 

Efforts to elucidate the mechanism of action of compounds 9b and 9c on the double-stranded nucleic acid unwind activity of the helicase of NS3 were carried out. Helicase assays using nucleic acid labeled with [γ-^32^P] ATP at its 5´end together with the T4 polynucleotide kinase, and 5´Cy3/3´BHQ2 labeled by fluorescence resonance energy transfer (FRET) were performed as described by Benarroch and co-workers and Boguszewska-Chachulska and co-workers [44,45], respectively. However, due to the low solubility of compounds in water and the increasing of ATPase activity in the presence of nucleic acids [46], it was not possible to assess the inhibitory potential of these compounds in the NS3 helicase activity. 

We also evaluated whether the compounds were able to suppress the proteolytic activity of DENV-2 NS3. Several studies have shown that the NS2B cofactor is required by the protease domain to form a proteolytic active domain [47,48]. Therefore, the NS3 protease domain linked to the NS2B cofactor was purified and the inhibition assay was performed as described by Leung and co-workers [11]. We did not observe any effect of compounds 9b and 9c against the NS3 protease activity even in the presence of the NS2b cofactor (data not shown). Therefore, the activity of the naphtoquinones 9b and 9c against the NS3 from DENV is specific and does not involve the proteolytic domain.

This study is one of the first to demonstrate the inhibitory effect of pyran naphthoquinone compounds on the ATPase domain of the DENV-2 NS3. Although, the mechanism of inhibition of nucleic acid unwinding has not been shown by the *in vitro* helicase assay, preliminary results in infected cells demonstrated a 3-fold decrease in intracellular viral RNA levels (data not shown) suggesting that these compounds act at the viral RNA replication level. Therefore, the mechanism of action of the 1,4-pyran naphthoquinones 9b and 9c could be related to the inhibition of the ATP hydrolysis and consequently block of the viral double strand RNA unwind that is the replication intermediate complex formed during the synthesis of the DENV genomic RNA. Mastrangelo and co-workers [49] showed that the anti-helminthic drug ivermectin inhibited the NS3 helicase activity of several flaviviruses, including Yellow Fever Virus (YFV), DENV and West Nile Virus and showed to be a selective inhibitor of the replication of these viruses in cell culture. It is noteworthy mentioning that a possible inhibitory effect at a sub-nanomolar concentration was observed only during YFV replication in cell culture.

Nevertheless, we cannot rule out that the mechanism by which these compounds inhibit the NS3 ATPase activity might be through blocking of the phosphate-binding region of the ATP molecule in the Walker A motif (manuscript in preparation).

Recently, it was demonstrated that an aglycon analogue of the antibiotic teicoplanin had a wide range activity against Flaviviruses targeting the initial steps of the viral replication cycle [50]. This compound inhibited DENV replication in Vero cells with an IC_50_ of 6.9μM and was considered a promising candidate for an anti-DENV drug. Although the naphthoquinones 9b and 9c most likely target post-entry steps of the DENV replication cycle and had a specific albeit less effective activity against the NS3 ATPase activity, the concentration of the 9c naphthoquinone required to inhibit 50% of the DENV replication in Vero cells was 20-fold lower when compared to the concentration of the aglycon analogue of the teicoplanin. Demonstrating the remarkable efficacy of the compounds identified in this study. The elucidation of the precise mode of action of these synthetic naphtoquinones against DENV replication will allow the development of a new class of anti-Dengue drugs.
